# Utilization of outreach immunization services among children in Hoima District, Uganda: a cluster survey

**DOI:** 10.1186/s13104-017-2431-1

**Published:** 2017-02-27

**Authors:** Paul Oryema, Juliet N. Babirye, Charles Baguma, Peter Wasswa, David Guwatudde

**Affiliations:** 1 School of Public Health, Makerere University College of Health Sciences, P.O. Box 7072, Kampala, Uganda; 2 African Field Epidemiology Network (AFENET), P.O. Box 12874, Kampala, Uganda

**Keywords:** Child, Immunization, Routine, Utilization, Outreach, Cluster survey, Uganda

## Abstract

**Background:**

The global vaccine action plan 2011–2020 was endorsed by 194 states to equitably extend the benefits of immunization to all people. However, gaps in vaccination coverage remain in developing countries such as Uganda. One of the strategies used to tackle existing inequities is implementation of outreach immunization services to deliver services to those with poor geographical access. However, reports of inconsistent use of these services prevail; therefore understanding the factors associated with use of these services is critical for improving service delivery. This study examined the factors associated with utilization of outreach immunization services among children aged 10–23 months in Hoima District, Uganda.

**Results:**

Overall, 87.4% (416/476) of the children had ever utilized outreach immunization services. Of these, 3.6% (15/416) had completed their entire immunization schedules from outreach immunization sessions. Use of outreach services was associated with reports that the time of outreach sessions was convenient [adjusted odds ratio (AOR) 2.9, 95% confidence interval (CI) 1.32–6.51], community mobilization was done prior to outreach sessions (AOR 4.9, 95% CI 1.94–12.61), the caretaker knew the benefits of childhood immunizations (AOR 2.1, 95% CI 1.30–4.42), and the caretaker was able to name at least four vaccine preventable diseases (AOR 3.0, 95% CI 1.13–7.88).

**Conclusions:**

Utilization of outreach immunization services in Hoima District was high but reduced with subsequent vaccine doses. Therefore, strategies targeted at retaining service users for the entire immunization schedule need to be developed and implemented. Such strategies could include health education emphasizing the benefits of childhood immunization.

## Background

Globally, vaccine preventable diseases (VPDs) account for over 1.5 million deaths among children under 5 years of age each year. Most of these deaths occur in developing countries such as Uganda. Improving utilization of immunization services is therefore essential to enhancing child survival with its associated economic benefits [1, 2]. However, immunization coverage in Uganda varies with different geographical locations and is lower in rural settings [1]. In Hoima, one of the rural Ugandan districts, coverage for DPT3 among children aged 12–23 months is administratively estimated at 72% for DPT3 and measles at 76%. These are all below the district and the national targets of 85% for DPT3 and 90% for measles [2] and may partly explain the frequent outbreaks of vaccine preventable diseases.

Many strategies have been used to improve and sustain utilization of routine childhood immunization services; one of which is establishing outreach immunization services. Outreach immunization services are used globally to engage vulnerable individuals and communities with limited geographical access to health facilities [3–8]. These outreach immunization services are particularly important in developing countries like Uganda, where 88% of its population resides in rural areas [9]. Outreach immunization services are usually conducted 5 km away from health facilities on a monthly basis in Uganda. Thus the targeted communities are expected to utilize them since they are within their reach; however there is limited evidence to suggest so.

Studies have reported varying use of other outreach health care services. For instance utilization of outreach dental care programs in India was reported as low as 28%, China 20%, and Spain 34% whereas in United States of America it was as high as 75 and 61% in United Kingdom [15–17]. Whereas studies have looked at utilization of other outreach health care services, to our knowledge none have examined outreach immunization services. This study therefore examined factors associated with utilization of outreach immunization services among children aged 10–23 months in Hoima District, Uganda.

## Methods

### Study area

This community based cross sectional study was conducted in Hoima District in mid-western Uganda between March and May 2013. Hoima District is 230 km from Kampala, the Capital City of Uganda. The district is one of the ten most populated districts in Uganda with an estimated population of 570,000 [10] (based on 2014 provisional census report) spread over an area of 5735.3 km^2^ (3612.17 km^2^ land and 2123.13 km^2^ covered by water (mostly Lake Albert). With a growth rate of 4.3% (2014 provisional census report), Hoima District has an estimated 106,000 children under 5 years of age and approximately 23,000 infants.

It has 54 public, private not for profit (PNFP) and private for profit (PFP) registered health facilities that provide health services. At the time of this study there were 124 estimated outreach vaccination posts that offer free routine immunization services to those with limited geographical access to static immunization services. All the public, PNFP and some PFP facilities provide static routine immunization services.

### Sample size determination

We sampled 476 households in 68 clusters (villages) using Bennett et al. [11] cluster survey sampling method with the following assumptions; a two-sided test with confidence interval of 95 and 5% level of significance, DPT3 coverage of 72% [1], design effect of 1.6, and intra-cluster correlation of 0.1.

### Sampling

At household level, caretakers of children aged 10–23 months were selected for inclusion in the study if they had lived in the study area for at least 10 months, and were 18 or more years old. One eligible caretaker-child pair were selected in each household. If there were more than one eligible child in a household, the youngest was selected and in cases of multiple births one child was selected using a table of random numbers. A child’s immunization status was assessed using immunization cards or their caretakers’ verbal report.

A three-stage cluster sampling technique was used. In the first stage, all 13 sub counties in the district were listed from which six sub counties of Buhanika, Kyabigambire, Buhimba, Kigorobya, Kitoba and Kiziranfumbi were selected using simple random selection method.

In the second stage all outreach vaccination posts and villages they serve were listed. Using computer generated random numbers, 68 clusters (villages) were selected without replacement.

At the third stage, eligible households in each cluster were selected by; identifying a random junction in the village. Then beginning with the first house, research assistants moved from house to house towards the centre of the village looking for eligible participants. In cases where a participant declined to participate, or was not home, the next household was considered for inclusion. This process continued till appropriate numbers of respondents were obtained in that village. Number of respondents per clusters were based on probability proportionate to size (PPS) based on number of eligible children (aged 10–23 months) in each cluster. Numbers of eligible children were obtained from village local council chairpersons’ mosquito nets distribution list.

### Data collection

Interviewer administered questionnaires were used to collect data on social demographic variables for child, caretaker and/or household heads, factors such as convenience of time of outreach sessions, community mobilization, caretakers’ knowledge on immunization, frequency of outreach sessions, health workers’ behaviors among others were also collected. The questionnaires were written in English and translated to Runyoro, the commonest local language used in the study area and then back translated to English to check for consistency of meaning.

### Data analysis

We entered data into Epi-Info 3.5.1 and exported to STATA 10 for cleaning, coding and analysis. Outcome variables were dichotomized into those who utilized (*received vaccination from an outreach immunization posts*) and those that did not utilize outreach immunization services. Level of utilization of outreach immunization services were determined by getting proportions of children who were vaccinated at an outreach immunization posts out of the entire children included in the study. One’s level of utilization was considered high when they had utilized outreach immunization services three or more times and low when less than three out of the five recommended routine immunization schedules.

In order to identify factors associated with utilization of outreach immunization services, we initially conducted univariate analysis, followed by backward stepwise multiple logistic regression at 95% confidence interval. Factors that were significant at bivariate level (p ≤ 0.05) were included in the multivariable regression model.

### Ethical approval

Ethical clearance was obtained from Makerere University School of Public Health Higher Degrees, Research and Ethics Committee. Study participants provided written informed consent before the interview.

## Results

A total of 476 primary caretakers of children aged 10–23 months were approached for study inclusion and all were enrolled into the study. Study respondents were aged 18–66 years, with a mean age of 27.8 years (SD = 8.7) and a median age of 26 years. The majority were females (94.1%, 448/476), mothers to the children (90%, 426/476), living with their partners (80.3%, 382/476), engaged in subsistence farming (73.3%, 349/476) and 59.9% (285/476) had seven years of formal education. The children considered in this study had a mean age of 18 months (SD = 5.2) and a median age of 19 months. Half (52.5%, 250/476) of the children were females and most had been delivered in health facilities (81%, 386/476), Table 1.

**Table 1:** Socio-demographic characteristics of the caretaker and child

Variables	Frequency n = 476	Percent
Sex of caretaker		
Male	28	5.9
Female	448	94.1
Age of caretaker		
18–25	233	49.0
26–35	162	34.0
Above 35	81	17.0
Marital status of caretaker		
Never married	23	4.8
Married	148	31.1
Cohabiting	234	49.2
Divorced/separated/widow	71	14.9
Religion of caretaker		
Catholic	191	40.1
Protestant	206	43.3
Others	79	16.6
Years of education of caretaker		
No education	22	4.6
7 years	285	59.9
12 years	148	31.1
14 or more years	21	4.4
Occupation of caretaker		
Farming	349	73.3
Small scale business	51	10.7
Others	76	16.0
Years of formal education of household head		
7 years	210	46.4
12 years	162	35.8
14 years or more	25	5.5
Don’t know	56	12.4
Age of child in months		
10–14	140	29.4
15–19	115	24.2
20–24	221	46.4
Sex of child		
Male	226	47.5
Female	250	52.5
Place of delivery of child		
Home	90	18.9
Health facility	386	81.1

### Use of outreach services

Overall 87.4% (416/476) of the children were vaccinated from an outreach immunization post. Of these, 30.5% (127/416) had used those services once, 34.6% (144/416) twice, 20.7% (86/416) thrice and only 3.6% (15/416) had used these services for all the required five immunization schedules.

The majority (97%, 461/476) of the respondents knew the benefits of immunizing children; however, only 19% (90/476) could mention at least four vaccine preventable diseases. Those who knew when immunization starts were 86% (411/476) and when it is completed were 56% (267/476). Only 29% (136/476) knew the number of times a child should be taken for routine immunization and 56% (266/476) were able to name the last vaccine or disease vaccinated against to complete immunization schedules.

### Factors independently associated with utilization

Use of outreach services was independently associated with reports that the time of outreach sessions was convenient [adjusted odds ratio (AOR) 2.9, 95% confidence interval (CI) 1.32–6.51] and that community mobilization was done prior to outreach sessions (AOR 4.9, 95% CI 1.94–12.61). Utilization of outreach services was also higher among those who knew the benefits of childhood immunization (AOR 2.1, 95% CI 1.30–4.42) and those who could mention at least four vaccine preventable diseases (AOR 3.0, 95% CI 1.13–7.88). The factors that were not statistically associated with utilization of outreach immunization services were: maternal age, religion, sex of child, number of children, educational level, and occupation of caretakers, see Table 2.

**Table 2:** Utilization of outreach immunization services

Variable	Total n = 476	Utilized outreaches (n, %)	Crude OR (95% CI)	Adjusted OR (95% CI)^a^
Age of caretakers				
18–25	233	199 (85.4)	1.0	1.0
26–35	162	146 (90.1)	2.1 [1.07–4.26]	2.1 [0.97–4.37]
Above 35	81	74 (91.4)	2.1 [0.86–5.29]	1.9 [0.71–4.99]
Completed years of education of caretakers				
No education	22	21 (95.5)	1.0	
7 years	285	252 (88.4)	0.5 [0.06–3.52]	
12 years	148	129 (87.2)	0.3 [0.04–2.71]	
14 or more years	21	14 (66.7)	0.1 [0.01–1.09]	
Occupation of caretakers				
Subst. farming	349	313 (89.7)	1.0	1.0
Traders	51	43 (84.3)	0.6 [0.24–1.43]	0.8 [0.29–2.09]
Others	76	60 (78.9)	0.4 [0.19–0.75]	0.4 [0.20–0.96]
Number of children				
One	121	104 (86.0)	1.0	
Two	120	101 (84.2)	0.9 [0.42–1.79]	
Three	74	65 (87.3)	1.5 [0.57–3.73]	
Four and over	161	146 (90.7)	2.1 [0.93–4.66]	
Child birth order				
First born	155	132 (85.2%)	1.0	1.0
Second	102	89 (87.3%)	1.2 [0.58–2.63]	1.2 [0.56–2.49]
Third	73	62 (84.9%)	1.3 [0.57–3.18]	1.0 [0.44–2.18]
Fourth and above	146	133 (91.1%)	2.3 [1.02–4.93]	1.8 [0.85–3.74]
Sex of child				
Male	226	196 (86.7)	1.0	1.0
Female	250	220 (88.0)	1.2 [0.69–2.17]	1.6 [0.82–2.97]
Knew benefit of child immunization				
No	461	406 (88.1)	1.0	1.0
Yes	15	10 (66.7)	0.2 [0.07–0.69]	2.1 [1.30–4.42]
Named at least four VPDS				
Yes	386	333 (86.3)	1.0	1.0
No	90	83 (92.2)	2.0 [0.78–4.58]	3.0 [1.13–7.88]
Time for outreach session				
Not convenient	59	46 (78.0)	1.0	1.0
Convenient	417	370 (88.7)	2.7 [1.36–5.51]	2.9 [1.32–6.51]
Vaccine unavailability				
No	354	317 (89.5)	1.0	1.0
Yes	122	99 (81.1)	0.5 [0.28–0.92]	0.6 [0.32–1.23]
Community mobilized for outreach				
No	33	23 (69.7)	1.0	1.0
Yes	443	393 (88.7)	4.2 [1.85–9.30]	4.9 [1.94–2.61]

^a^Adjusted for knowing the benefits of immunization, naming at least four VPDs, community mobilization and convenience of time of outreach sessions

## Discussion

This study examined the factors associated with utilization of outreach immunization services among children age 10–23 months in Hoima District, Uganda. Overall, more than three quarters of all sampled children had ever utilized outreach immunization services although this proportion reduced with subsequent vaccine doses. After multivariable analysis the factors independently associated with utilization of outreach immunization services were reports of convenient time of outreach sessions, community mobilization prior to outreach sessions, knowing the benefits of immunization, and being able to name at least four vaccine preventable diseases.

The high level of utilization of outreach immunization services seen in Hoima has been similarly reported by other studies on HIV/AIDS service usage [12–14]. According to Uganda’s routine childhood immunization schedule; BCG and polio0 are given at birth; polio and pentavalent vaccines are then given at 6, 10, and 14 weeks; and lastly measles is given at 9 months [15]. Overall, the mother–child pair has five recommended visits to the routine immunization site. However, our study shows that a third of these children had received vaccination at outreach immunization sessions for three or more times and only 4% for the entire routine immunization schedules. Outreach immunization sessions bring services closer to communities with limited geographical access to health facilities to enable the completion of the immunization schedule. Therefore, it is expected that communities served by these outreaches will utilize them since they are within their reach. Thus our study finding was surprising and could be due to irregularity of outreach sessions as reported by other researchers [16], or an indication of poor quality of services as shown by the high dropout rate in the district (23.7% for DPT3; acceptable rate is 10%) [17], similar to that seen in other settings [1, 18, 19]. Additionally, 71% of caretakers in our study did not know the recommended schedules to complete routine childhood immunization further compounding the level of dropout. This low level of awareness among caretakers may reflect poor communication between service providers and caretakers.

Respondents who reported that the time outreach immunization sessions were offered was convenient more often utilized these services compared to those who reported otherwise. Similar findings have been reported elsewhere [8, 16]. Implying that scheduling of outreach immunization sessions should consider the time and nature of activities targeted communities are engaged in, to maximize their likelihood of utilizing them. In our study, the majority of the respondents were practicing subsistence farming, which means during morning and evening hours they are likely to be engaged in agricultural activities, thus limiting their participation in immunization sessions which are often scheduled in the mornings. Therefore the time for conducting outreach immunization sessions should be flexible as reported in Vietnam [3]. Having flexible times for outreach immunization sessions can create more opportunities for caretakers particularly because childhood immunization does not provide apparent immediate benefits and thus caretakers may not be motivated to prioritize it amidst other competing work priorities [5].

Over 88% of caretakers reported that their communities were mobilized prior to outreach sessions and this impacted positively on utilization of outreach immunization services. Studies that have used community mobilization as an intervention to improve immunization uptake have also reported similar findings [14]. Effective community mobilization makes populations more responsive to immunization services and this helps sustain its utilization [20]. However, community mobilization requires all stakeholders including: local government, service providers, community leaders and village health teams [21] coordinated by district health teams [22]. With targeted community mobilization, there can be drastic improvement in utilization of not only outreach immunization services but routine immunization services in general.

In Hoima District, caretakers’ knowledge on the benefits of childhood immunization was associated with better utilization of immunization services. Similar findings have also been reported in India, China and Ethiopia [8, 23, 24]. In our study, caretakers who were able to name at least four vaccine preventable diseases were more likely to have utilized outreach immunization services compared to those who could not. This is in agreement with studies in Ghana and Ethiopia [25, 26]. However, it is possible that the knowledge may be a result of, rather than the reason for utilization of immunization services. Nevertheless, prevailing data indicates that knowledge on immunization related issues is a good predictor of uptake of immunization services [25, 27, 28]. Therefore, deliberate efforts geared towards educating communities on the benefits of immunizations is critical in increasing utilization of outreach immunization services.

### Methodological considerations

This study was conducted in Hoima which consists of mostly rural residents 82% [10]. Our results therefore may have implications for immunization programs in similar settings of Sub-Saharan Africa. In interpreting these results however, some limitations of the study design should be considered. First, a differential error in measurement could have arisen if those who did not use outreach immunization services were more likely to report overuse of these services because of perceived unacceptability of sub-optimal use of the services. However, several studies done in other settings using a similar study design have obtained comparable findings [8, 19, 22, 29, 30]. Secondly, we assessed utilization of outreach services at a specific point in time, that is, 10–23 months prior to the survey. This overlooks the varying nature of use of these services over the years since current use of outreach services does not necessarily predict previous or future patterns of service usage. Nevertheless, this study selected survey participants within the community, essentially eliminating the selection bias that could have arisen if the participants were obtained at immunisation facilities.

## Conclusions

Most studies on routine child immunization services usually focus on those services provided at health facilities. Our analysis of utilization of outreach immunization services in this rural setting shows that the usage of these services is not optimal since the proportions of those that had used them declined with subsequent vaccine doses to less than 5% of total study subjects. The factors associated with utilization of these outreach immunization services in Hoima included reports of convenient time of outreach sessions, community mobilization prior to the session, being able to name at least four vaccine preventable diseases and knowing the benefits of child immunization. Strategies that aim to retain service users for the entire immunization schedule need to be developed and implemented. Such strategies could include health education emphasizing the benefits of childhood immunization, community mobilization prior to outreach sessions as well as deciding the time for outreach sessions in consultation with the community.

## Abbreviations

AFENET: African Field Epidemiology Network
AIDS: acquired immunodeficiency syndrome
AOR: adjusted odds ratio
BCG: Bacille Calmette–Guérin
CDC: Centers for Disease Control and Prevention
CI: confidence interval
DPT: diphtheria pertusis tetanus
HIV: human immunodeficiency virus
MPH: Master of Public Health
PFP: private for profit
PNFP: private not for profit
PPS: probability proportionate size
SD: standard deviation
USAID: United States Agency for International Development
VPDs: vaccine preventable diseases
